# In Vitro Preventive Effect and Mechanism of Action of *Weissella cibaria* CMU against *Streptococcus mutans* Biofilm Formation and Periodontal Pathogens

**DOI:** 10.3390/microorganisms11040962

**Published:** 2023-04-07

**Authors:** Mi-Sun Kang, Geun-Yeong Park, A-Reum Lee

**Affiliations:** R&D Center, OraTicx, Inc., Seoul 04782, Republic of Korea; gypark@oraticx.com (G.-Y.P.); lar925@oraticx.com (A.-R.L.)

**Keywords:** biofilm, gene expression, human gingival fibroblast, metalloproteinase, periodontopathogen, probiotics, pro-inflammatory cytokine, *Streptococcus mutans*, *Weissella cibaria* CMU

## Abstract

In this study, we evaluated the in vitro anti-biofilm, antibacterial, and anti-inflammatory activity of *Weissella cibaria* CMU (CMU), an oral probiotic, against periodontopathogens. Compared to other oral probiotics, CMU showed a superior inhibitory effect on the biofilm formation and growth of *Streptococcus mutans* on orthodontic wires and artificial teeth (*p* < 0.05). CMU exerted potent antibacterial effects against *S. mutans* and *Porphyromonas gingivalis* according to a line test. In human gingival fibroblasts (HGFs) stimulated by *P. gingivalis, Fusobacterium nucleatum*, or *Prevotella intermedia*, CMU suppressed the gene expression of pro-inflammatory cytokines [interleukin (IL)-6, IL-1β, IL-8, and tumor necrosis factor-α] in a dose-dependent manner (*p* < 0.05). CMU restored the production of the tissue inhibitor of metalloproteinase-1 following its inhibition by *P. gingivalis*, and it suppressed the expression of matrix metalloproteinase (MMP)-1 and -3 induced by periodontopathogens (*p* < 0.05). Moreover, CMU needed direct contact with HGFs to exert their anti-inflammatory function, indicating that they act directly on gingival cells to modulate local inflammation. Our preclinical study provides evidence for the potential benefits of topical CMU treatments in preventing the development of caries and periodontitis caused by the dysbiosis of the dental plaque microbiome.

## 1. Introduction

Dental caries and periodontal disease are the most common oral diseases worldwide [1,2,3]. Dental caries particularly occurs in childhood due to unhealthy oral hygiene habits, eating habits, and oral bacterial alterations which are considered risk factors for caries [4]. In the formation of dental caries, the tooth enamel is damaged and tooth decay is caused by acids produced by the bacterial breakdown of sugars [5]. Plaque is a major direct cause of dental caries and is characterized by a persistent biofilm. Therefore, the prevention of dental caries can be achieved by inhibiting the plaque-related biofilm formation due to various cariogenic bacteria including *Streptococcus mutans* [5].

Periodontitis—a chronic disease that destroys the alveolar bone supporting the gums and teeth through exacerbated local inflammation—is one of the leading causes of tooth loss in adults [2,6]. Periodontitis is caused by periodontopathogens, such as *Porphyromonas gingivalis* [7,8]. Chronic inflammation due to repeated bacterial infection promotes the secretion of pro-inflammatory cytokines and matrix metalloproteinases (MMPs) [9]. Interleukin (IL)-6, IL-1β, IL-8, and tumor necrosis factor (TNF)-α are prominent pro-inflammatory cytokines associated with periodontal tissue destruction [10,11].

MMPs are enzymes that can destroy the connective tissue of the gums upon overactivation [12,13]. Collagenases (MMP-1, -8, and -13), gelatinase (MMP-2 and -9), and stromelysin-1 (MMP-3) have all been implicated in periodontitis [12,13,14]. Among the MMPs, MMP-1 can either directly degrade collagen or activate the fibrotic protease cascade to destroy connective tissue [14]. MMP-3-mediated collagen degradation is a major pathway for connective tissue destruction through extracellular matrix degradation and MMP-1 activation [14]. The release of inflammatory mediators, such as pro-inflammatory cytokines and MMPs, alters the state of the connective tissue and bone metabolism, leading to the destruction of the periodontal ligament and the resorption of alveolar bone. Therefore, inhibiting the expression of these inflammatory mediators is considered a promising approach to the prevention of periodontal disease.

Gingivitis is an early inflammatory stage of periodontitis and can be treated with scaling and plaque removal, while bone damage in periodontitis is irreversible. Therefore, gingivitis prevention may be the most effective strategy to avoid the occurrence of periodontitis. Currently, mechanical removal methods such as scaling and root planing are used to treat periodontitis, while antibiotics and antibacterial agents such as tetracycline, minocycline, doxycycline, metronidazole, and chlorhexidine are used as adjuvants to sustain the temporary effect of mechanical removal [15,16]. However, the efficacy of this approach is limited by various side effects, such as increased antibiotic resistance, gastrointestinal hypersensitivity, and toxicity [17,18,19]. Therefore, safe and effective alternative therapies for the prevention and treatment of periodontal disease are urgently needed.

Probiotics have recently received considerable attention as a safe and promising approach to preventing oral diseases [20,21,22,23,24,25,26,27,28,29,30,31,32,33,34,35,36,37,38]. Probiotics are defined as “living microorganisms which, when administered in adequate amounts, confer a health benefit on the host” [39]. Oral probiotics work by regulating plaque formation and preventing the disruption of microbial homeostasis [20,21,22]. Several oral probiotics, including *Lactobacillus reuteri*, *Streptococcus salivarius*, and *Weissella cibaria*, have been reported to attenuate dental caries, periodontitis, and halitosis [23,24,25,26,27,28,29,30,31,32,33,34,35,36,37,38]. In addition to probiotics, postbiotics and paraprobiotics are being studied as adjunctive therapies for non-surgical scaling and root planing in an effort to reduce periodontal microorganisms. Postbiotics are defined as the “preparation of inanimate microorganisms and/or their components that confers a health benefit on the host” [40]. Postbiotics are supposed to be a natural alternative to traditional chemical substances like chlorhexidine. Paraprobiotics are heat-killed (tyndallized) probiotics and together with postbiotics, they are classified as non-viable probiotics [40]. Several studies have reported various alternative treatments, such as postbiotics-based antimicrobial gel [41], paraprobiotics-based toothpaste, mouthwash containing genus *Lactobacillus*, *Bifidobacterium*-based toothpaste and mouthwash [42], and probiotics-based toothpaste or chewing gum [43], all of which have been applied to clinical trials.

Most *Lactobacillus* spp. are representative lactic acid bacteria that generally produce a strong acid that can be neutralized by the buffering function of saliva in the oral cavity of healthy people, but this has the potential to cause tooth decay [44]. On the other hand, *W. cibaria* can prevent caries because it inhibits biofilm formation by *S. mutans* [32] and has a higher ecological pH than Lactobacilli strains [30]. In addition, our previous work showed that when *W. cibaria* CMU tablets were consumed for 8 weeks, *W. cibaria* colonized the oral flora and eliminated the risk of dental caries due to acid production [36]. *W. cibaria* CMU and CMS1 are oral probiotic strains commercially available in Korea [33]. These lactic acid bacteria are isolated from the saliva of children aged 4 to 7 years old who have little supragingival plaque and no oral diseases, including dental caries [30,33]. Many previous studies have demonstrated the beneficial effects of *W. cibaria* CMU on oral health [30,31,32,33,34,35,36,37,38]. A comparative study with other oral probiotics also reported that *W. cibaria* CMU had potent inhibitory effects against *S. mutans* biofilm formation as well as *P. gingivalis* and *F. nucleatum* proliferation [32].

Recently, dead *W. cibaria* CMU was reported to be potentially effective in preventing periodontitis by downregulating the gene expression of *P. gingivalis* lipopolysaccharide (LPS)-induced pro-inflammatory cytokines and MMPs in human gingival fibroblasts (HGFs) [37]. However, the effects of live *W. cibaria* CMU on the expression and production of periodontal pathogen-stimulated pro-inflammatory cytokines and MMPs in HGFs, as well as on the regulation of biofilm formation by *S. mutans* on orthodontic wires and artificial teeth, have not yet been investigated. Therefore, the present study aimed to determine the in vitro efficacy of live *W. cibaria* CMU on the inhibition of cariogenic and periodontal pathogens.

## 2. Materials and Methods

### 2.1. Bacterial Strains and Growth Conditions

Five oral pathogens (*S. mutans* Ingbritt, *S. mutans* KCTC 3065, *Fusobacterium nucleatum* KCTC 2488, *P. gingivalis* ATCC 33277, and *Prevotella intermedia* ATCC 25611) were used in this study. *S. mutans* was grown in brain heart infusion broth (BHI broth; MB cell, Difco, Kisan Bio, Seoul, Korea) for 16 h at 37 °C under aerobic conditions. *F. nucleatum*, *P. gingivalis*, and *P. intermedia* were grown in tryptic soy broth (TSB) hemin menadione (MB cell) supplemented with 5 μg/mL hemin (MB cell) and 0.5 μg/mL menadione (MB cell) under anaerobic conditions (AnaeroPack-Anaero, Mitsubishi Gas Chemical, Tokyo, Japan) at 37 °C for 48 h.

For comparison of oral probiotic activities, *W. cibaria, L. reuteri*, *Lactobacillus paracasei*, *Lactobacillus gasseri*, and *S. salivarius* were used. We procured the commercial strains *W. cibaria* CMU (oraCMU^®^) and CMS1 (oraCMS1^®^) from OraTicx (Seoul, Korea). *L. reuteri*, *L. paracasei*, and *L. gasseri* were isolated from Korean commercial oral probiotic products using Mann, Rogosa, and Sharpe (MRS) agar (Difco, Detroit, MI, USA), and *S. salivarius* was isolated from global oral probiotic products using tryptic soy agar (MB cell). All bacterial strains were identified through 16S rRNA sequence analysis. *W. cibaria* and *Lactobacillus* spp. were grown aerobically in MRS broth (Difco), and *S. salivarius* was grown in TSB (MB cell), all at 37 °C for 16 h.

### 2.2. Evaluation of Oral Probiotics on S. mutans Biofilm Formation

#### 2.2.1. Effects of Oral Probiotics on *S. mutans* Biofilm Formation on Orthodontic Wires

To determine the effects of oral probiotics on biofilm formation by *S. mutans*, we performed a tube wire test as previously described with minor modifications [30]. Briefly, equal amounts (5 × 10^6^ CFU/mL) of *S. mutans* and each oral probiotic were cultivated in a tube containing 30 mL of the test medium {pH 6.5; equal amounts of BHI and MRS with 5% sucrose, 0.5% yeast extract [MB cell], and 0.1 M MES [2-(N-Morpholino) ethanesulfonic acid monohydrate; MB cell]}. One orthodontic wire with a length of 4 cm and a diameter of 0.8 mm (Remanium, Dentaurum, Pforzheim, Germany) was suspended from a conical tube and immersed in the test medium. After gentle shaking (50 rpm) at 37 °C for 24 h, the weight of the *S. mutans* biofilm formed on each wire was measured. *S. mutans* inoculated alone was used as a control. We confirmed the dose-dependent effects of *W. cibaria* in 10-fold serial dilutions relative to the *S. mutans* concentration.

To measure the effects of oral probiotics on the growth of *S. mutans* after orthodontic wire removal, all cultures were serially diluted and inoculated onto Mitis Salivarius Bactiracin (MSB) agar (Difco).

#### 2.2.2. Effects of Oral Probiotics on *S. mutans* Biofilm Formation on Artificial Teeth

We assessed the effects of oral probiotics on *S. mutans* biofilm formation on artificial teeth as previously described, with minor modifications [45]. In summary, 1.5 mL of the test medium (see Section 2.2.1.) was added to a 24-well plate containing resin-based artificial teeth (VIPI-DENT plus, Madespa, Toledo, Spain) and inoculated with equal amounts (5 × 10^6^ CFU/mL) of *S. mutans* and oral probiotics. After culturing for 24 h at 37 °C, the supernatant was completely removed and wells containing teeth were rinsed twice with phosphate-buffered saline. To measure the amount of biofilm formed, each tooth was stained with 0.1% safranin (BD Biosciences, Sparks, MD, USA) for 15 min, rinsed with distilled water three times, and treated with 30% acetic acid to release bound safranin from the stained cells; the absorbance of the solution was measured at 530 nm. *S. mutans* inoculated alone was used as a control. We confirmed the dose-dependent effects of *W. cibaria* using 10-fold serial dilutions relative to the *S. mutans* concentration.

### 2.3. Evaluation of Oral Probiotic Antagonism against Oral Pathogens

We determined the antagonistic activity between oral probiotics and pathogens using line tests (a conventional antagonism test) as previously described, with minor modifications [46]. In summary, 20 μL *S. mutans* culture diluted to 10^6^ CFU/mL was first dropped onto agar mixed with equal amounts of BHI and MRS or BHI alone and allowed to flow vertically. *P. gingivalis* was inoculated on TSB hemin menadione agar containing 5% (*v*/*v*) sterile defibrinated sheep blood (MB cell) at 20 μL to achieve 10^7^ CFU/mL. After drying the pathogens, equal amounts and 10-fold dilutions of oral probiotics were vertically dropped across the pathogens from the edge of the plate. Agar plates for *S. mutans* and *P. gingivalis* were incubated aerobically and anaerobically at 37 °C for 3–7 d, respectively.

### 2.4. Evaluation of Oral Probiotic Efficacy in Preventing the Impact of Periodontal Pathogens

#### 2.4.1. Antioxidant Assay

The antioxidant activity of oral probiotics was tested with cell-free supernatants (CFSs) and evaluated using a 2,2-diphenyl-1-picrylhydrazyl (DPPH) free radical scavenging assay (Sigma-Aldrich, St. Louis, MO, USA), as previously described, with some modifications [47]. To prepare the bacterial CFSs, cells were removed by centrifugation (5000× *g*, 10 min, 4 °C) and the CFS was filtrated (0.22 μm; JET BIOFIL, Guangzhou, China). We mixed 400 µL of CFS and 800 μL of freshly prepared DPPH solution (0.2 mM in ethanol), which we left to react for 25 min at 25 °C and then centrifuged (5000× *g*, 5 min, 25 °C). We transferred 200 µL of the supernatants to 96-well plates. The mixture of MRS broth and DPPH was used as a blank treatment. The level of scavenged DPPH was measured at 517 nm using a microplate reader (VersaMax, Molecular Devices, San Jose, CA, USA).

The scavenging ability (%) was defined as follows: 100 × [optical density at 517 nm (OD_517_) blank − (OD_517_) sample]/(OD_517_) blank.

#### 2.4.2. Human Gingival Fibroblast Culture

HGFs (Lifeline Cell Technology, Walkersville, MD, USA) were provided by the Laboratory of Oral Anatomy (School of Dentistry, Wonkwang University, Iksan, Korea) and grown in Dulbecco’s modified Eagle’s medium (DMEM; Gibco, Thermo Fisher Scientific, Gaithersburg, MD, USA), supplemented with 10% heat-inactivated fetal bovine serum (Gibco) and 1% antibiotic–antimycotic solution (GenDepot, Katy, TX, USA) at 37 °C in a humidified atmosphere containing 5% CO_2_. Experiments were carried out on HGFs of passages 3 to 9. The cells were sub-cultured and plated at 80% confluency. Serum and antibiotic-free DMEM were used for the co-culture of HGFs and live bacteria.

#### 2.4.3. Bacterial Challenge

Bacterial cultures were harvested (5000× *g*, 10 min, 4 °C), washed with PBS, and resuspended in DMEM. The OD was measured at 600 nm (OD_600_) and the suspension was diluted to obtain an OD of 0.5, corresponding to 5 × 10^8^ CFU/mL. The relationship between OD_600_-CFU counts for each strain was obtained by extrapolating the CFU/mL using a preset standard curve. To examine the anti-inflammatory effects of oral probiotics, HGFs were seeded in 6-well plates at 2 × 10^5^ cells/well. After 24 h, the medium was changed to serum and antibiotic-free DMEM. For mRNA analysis, HGFs were pre-treated with oral probiotics at a multiplicity of infection (MOI) of 1:100 for 30 min. Periodontal pathogen infection was performed for over 4 h with the same amount as the probiotics. For enzyme-linked immunosorbent assay (ELISA) analysis, cells were pre-treated with oral probiotics for 30 min and cultured with periodontal pathogens for 24 h. Various doses of *W. cibaria* CMU (0.1, 1, and 10) relative to periodontal pathogen infection rates were also tested. To determine the effect of direct contact between *W. cibaria* CMU and host cells, *W. cibaria* CMU was separated from HGFs and periodontal pathogens in the co-culture system using a SPLInsert™ (pore size 0.4 μm; SPL Life Sciences, Pocheon, Korea) for 4 h or 24 h.

#### 2.4.4. Reverse Transcription (RT)-Quantitative Polymerase Chain Reaction (qPCR)

Total RNA was extracted with TRIzol^®^ reagent (Ambion, Life technologies, Carlsbad, CA, USA) according to the manufacturer’s instructions and quantified spectrophotometrically. The isolated RNA (500 ng) was reverse transcribed using a PrimeScript RT Reagent kit (Takara Bio, Shiga, Japan). Quantitative PCR was performed on a Rotor-Gene Q system (Qiagen, Hilden, Germany) using a PowerUp SYBR GreePCR Master Mix (Applied Biosystems, Thermo Fisher Scientific) with the following reaction conditions: initial denaturation at 95 °C for 2 min, followed by 50 cycles at 95 °C for 15 s and 60 °C for 1 min. The primer sequences were as follows: human IL-6 forward (F), 5′-AGACAGCCACTCACCTCTTCAG-3′ and reverse (R), 5′-TTCTGCCAGTGCCTCTTTGCTG-3′; human IL-1β F, 5′-GGCAATGAGGATGAC-TTGTTCT-3′ and R, 5′-CTGTAGTGGTGGTCGGAGATTC-3′; human IL-8 F, 5′-ATG-ACTTCCAAGCTGGCCGTGGCT-3′ and R, 5′-TCTCAGCCCTCTTCAAAAACTTCTC-3′; human TNF-α F, 5′-AGCCCATGTTGTAGCAAACC-3′; and R, 5′-ATGAGGTACAGGCCCTCTGA-3′; human MMP-1 F, 5′-GCTAACCTTTGATGCTATAACTACGA-3′ and R, 5′-TTTGTGCGCATGTAGAATCTG-3′; human MMP-3 F, 5′-CAGTTTGCTCAGCCTATCCA-3′ and R, 5′-TCACATCTTTTTCGAGGTCGT-3′; human MMP-8 F, 5′-GTTCAGCAAGCATTTTCGTT-3′ and R, 5′-CACGGAGGACAGGTAGAATG-3′; human MMP-9 F, 5′-ATTTCTGCCAGGACCGCTTC-3′ and R, 5′-TCATAGGTCACGTAGCCCACT-3′; human TIMP-1 F, 5′-TGGACTCTTGCACATCACTACCTGC-3′ and R, 5′-AGGCAAGGTGACGGGACTGGAA-3′; and human GAPDH F, 5′-AGCCACATCGCTCAGACAC-3′ and R, 5′-GCCCAATACGACCAAATCC-3′. Relative RNA expression was determined using the 2^–ΔΔCT^ method, and relative gene expression was normalized to that of *GAPDH*.

#### 2.4.5. ELISA Analysis

The concentrations of secreted inflammatory mediators were quantified using human-specific ELISA kits (DuoSet system, R&D Systems, Minneapolis, MN, USA). The 96-well plates were coated with anti-human MMP-1, MMP-3, MMP-8, MMP-9, or TIMP-1 monoclonal antibodies at 4 °C overnight. All assays were performed according to the manufacturer’s instructions, and the level of each inflammatory mediator was determined using the standard curve prepared for each assay. The optical density at 450 nm was measured for each well using the microplate reader, with wavelength correction set at 540 nm.

#### 2.4.6. Cell Viability Assay

To measure cell viability after treatment with live oral probiotics, we used a viability assay kit (Cellrix, MediFab, Seoul, Korea). HGFs were seeded on 96-well plates at a density of 10^4^ cells/well in only DMEM or DMEM containing 2% FBS. The cells were treated with various concentrations of oral probiotics (MOI = 0.1, 1, 10, and 100) for 24 h at 37 °C in a 5% CO_2_ atmosphere. The medium was then carefully replaced with a fresh medium containing water-soluble tetrazolium-8 (WST-8) salt solution, and the plates were incubated at 37 °C in a 5% CO_2_ atmosphere for 4 h. Cell viability was measured at 450 nm using the microplate reader.

### 2.5. Statistical Analysis

The results are presented as the mean ± standard deviation of triplicate measurements. Statistical analyses were performed using SPSS Statistics version 21.0 for Windows (IBM, Armonk, NY, USA). A one-way analysis of variance (ANOVA) with Duncan’s multiple range test was used to compare the differences between group means. Statistical significance was set at *p* < 0.05.

## 3. Results

### 3.1. In Vitro Beneficial Effects of Oral Probiotics on S. mutans Biofilm Formation

#### 3.1.1. Antibiofilm Activity against *S. mutans* on Orthodontic Wires

**Figure 1:**
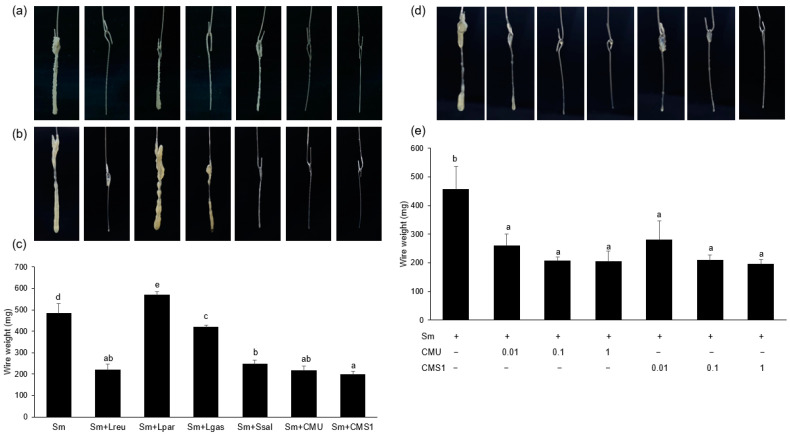
Effect of oral probiotics on biofilm formation by *S. mutans* KCTC 3065 (**a**) or *S. mutans* Ingbritt (**b**) on orthodontic wires. (**c**) Comparison of biofilm weight. (**d**) Dose-dependent effects of *W. cibaria* CMU and CMS1. (**e**) Comparison of biofilm weight according to the dose-dependent effects of *W. cibaria* CMU and CMS1. Sm, *S. mutans* Ingbritt; Lreu, *L. reuteri*; Lpar, *L. paracasei*; Lgas, *L. gasseri*; Ssal, *S. salivarius*; CMU, *W. cibaria* CMU; CMS1, *W. cibaria* CMS1. Data are presented as the mean ± standard deviation. Different letters indicate significant differences at *p* < 0.05.

We performed a tube wire test to determine the effects of oral probiotics against *S. mutans* antibiofilm formation on orthodontic wires. *L. reuteri*, *W. cibaria* CMU, and CMS1 strongly inhibited *S. mutans* biofilm formation, whereas other commercial oral probiotics had little inhibitory effects (Figure 1a–c). Both *W. cibaria* CMU and CMS1 inhibited *S. mutans* biofilm formation and growth in a dose-dependent manner (Figure 1d,e). The growth of *S. mutans* was inhibited by 99.99% by both *W. cibaria* CMU as well as CMS1 (*p* < 0.05) (Table 1).

#### 3.1.2. Antibiofilm Activity against *S. mutans* on Artificial Teeth

**Figure 2:**
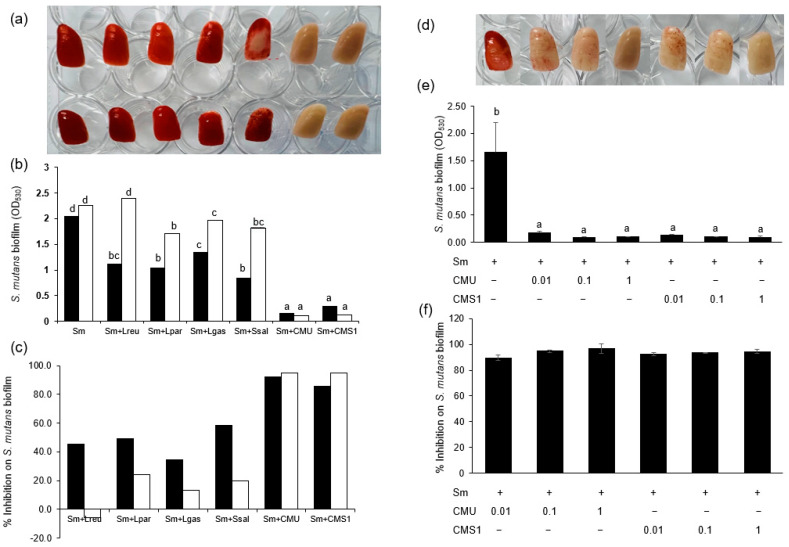
Effect of oral probiotics on biofilm formation by *S. mutans* on artificial teeth. (**a**) Effect of oral probiotics on biofilm formation compared between *S. mutans* KCTC 3065 (upper teeth) and *S. mutans* Ingbritt (lower teeth). (**b**) Comparison of *S. mutans* biofilm density. (**c**) Comparison of inhibition rates between oral probiotics. Equal amounts (5 × 10^6^ CFU/mL) of *S. mutans* and oral probiotics were inoculated. Black bar, *S. mutans* KCTC 3065; white bar, *S. mutans* Ingbritt. (**d**) Dose-dependent effects of *W. cibaria* CMU and CMS1. (**e**) Comparison of *S. mutans* biofilm density according to dose-dependent effects of *W. cibaria* CMU and CMS1. (**f**) Dose-dependent inhibition on *S. mutans* biofilm formation by *W. cibaria* CMU and CMS1. Sm, *S. mutans* Ingbritt; Lreu, *L. reuteri*; Lpar, *L. paracasei*; Lgas, *L. gasseri*; Ssal, *S. salivarius*; CMU, *W. cibaria* CMU; CMS1, *W. cibaria* CMS1. Data are presented as the mean ± standard deviation. Different letters indicate significant differences at *p* < 0.05.

We determined the effects of oral probiotics against *S. mutans* biofilm formation on resin-based artificial teeth. *W. cibaria* CMU and CMS1 inhibited *S. mutans* biofilm formation by 96.8% and 94.6%, respectively, whereas other commercial oral probiotics had no inhibitory effect (Figure 2a–c). Both *W. cibaria* CMU and CMS1 inhibited *S. mutans* biofilm formation on artificial teeth in a dose-dependent manner (Figure 2d–f).

### 3.2. Antibacterial Activity against S. mutans and P. gingivalis

**Figure 3:**
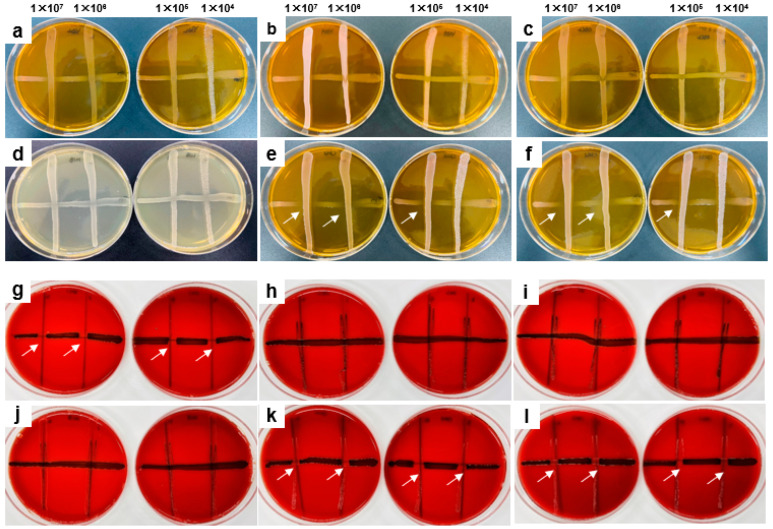
Comparison of antibacterial activities between oral probiotics against *S. mutans* Ingbritt (**a**–**f**) and *P. gingivalis* ATCC 33277 (**g**–**l**). Each horizontal line was inoculated with the tested pathogens and the vertical line was inoculated with 10-fold serial diluted oral probiotics. The white arrows indicate areas of poor pathogen growth and potent antibacterial activity of oral probiotics. (**a**,**g**) *L. reuteri*; (**b**,**h**) *L. paracasei*; (**c**,**i**) *L. gasseri*; (**d**,**j**) *S. salivarius*; (**e**,**k**) *W. cibaria* CMU; (**f**,**l**) *W. cibaria* CMS1.

We performed a line test to elucidate the antibacterial activity of oral probiotics against *S. mutans*, the representative cariogenic bacterium (Figure 3a–f), and *P. gingivalis*, a periodontopathic bacterium (Figure 3g–l). *W. cibaria* CMU and CMS1 showed a strong direct inhibition against both *S. mutans* (Figure 3e,f) and *P. gingivalis* (Figure 3k,l).

### 3.3. In Vitro Beneficial Effects of Oral Probiotics against the Impact of Periodontal Pathogens

#### 3.3.1. Antioxidant Activity

We measured the antioxidant activities of *L. reuteri*, *W. cibaria* CMU, and CMS1 CFSs. The highest antioxidant activity was observed in *W. cibaria* CMU (20.0 ± 4.5%), followed by *W. cibaria* CMS1 (16.7 ± 6.7%) and *L. reuteri* (15.2 ± 8.3%) (Table S1).

#### 3.3.2. Cytotoxic Effects of Oral Probiotics on HGFs

We evaluated the cytotoxic effects of live *L. reuteri*, *W. cibaria* CMU, and CMS1 according to the viability of HGFs. No cytotoxic effects were detected after the 24 h challenge with various bacterial concentrations in either test medium (Table 2).

#### 3.3.3. Inhibitory Effect of *W. cibaria* on Periodontopathogen-Induced Pro-Inflammatory Cytokine Expression

**Figure 4:**
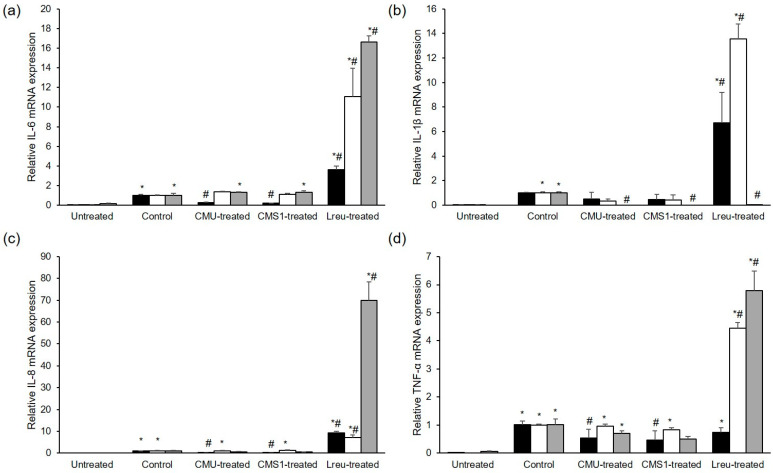
Comparison of the effects of oral probiotics on pro-inflammatory cytokine gene expression, including IL-6 (**a**), IL-1β (**b**), IL-8 (**c**), and TNF-α (**d**), stimulated by periodontopathogens. Black bar, *P. gingivalis*; white bar, *F. nucleatum*; gray bar, *P. intermedia;* CMU, *W. cibaria* CMU; CMS1, *W. cibaria* CMS1; Lreu, *L. reuteri*. Data are presented as the mean ± standard deviation. * *p* < 0.05, compared to the untreated group. ^#^ *p* < 0.05, compared to the control group.

**Figure 5:**
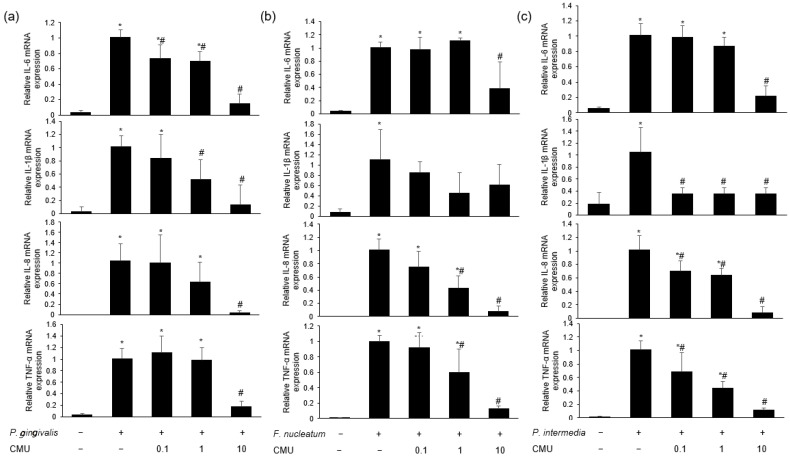
Dose-dependent effects of *W. cibaria* CMU on the mRNA expression of pro-inflammatory cytokines stimulated by *P. gingivalis* (**a**), *F. nucleatum* (**b**), and *P. intermedia* (**c**). HGFs were pre-treated with *W. cibaria* CMU for 30 min at various doses (0.1, 1, and 10) and then incubated with each periodontal pathogen (MOI = 100) for 4 h. CMU, *W. cibaria* CMU. Data are presented as the mean ± standard deviation. * *p* < 0.05, in comparison to the untreated group. ^#^ *p* < 0.05, in comparison to the control group.

Based on the above results indicating that *L. reuteri*, *W. cibaria* CMU, and CMS1 showed antibacterial activity against *P. gingivalis*, we compared the effects of these bacteria on the mRNA expression of pro-inflammatory cytokines. *L. reuteri* had no inhibitory effect on most pro-inflammatory cytokines (IL-6, IL-8, IL-1β, and TNF-α), and it dramatically promoted their expression compared to periodontal bacteria alone (*p* < 0.05) (Figure 4). We assessed the mRNA expression of pro-inflammatory cytokines in HGFs pre-treated with various concentrations of *W. cibaria* and stimulated by *P. gingivalis* (Figure 5a), *F. nucleatum* (Figure 5b), or *P. intermedia* (Figure 5c). *W. cibaria* CMU suppressed the mRNA expression of most pro-inflammatory cytokines in a dose-dependent manner compared to that in treatments with the periodontopathogen alone, and it showed a potent inhibitory effect at higher doses (*p* < 0.05).

#### 3.3.4. Inhibitory Effect of *W. cibaria* on Periodontopathogen-Induced MMP Expression

**Figure 6:**
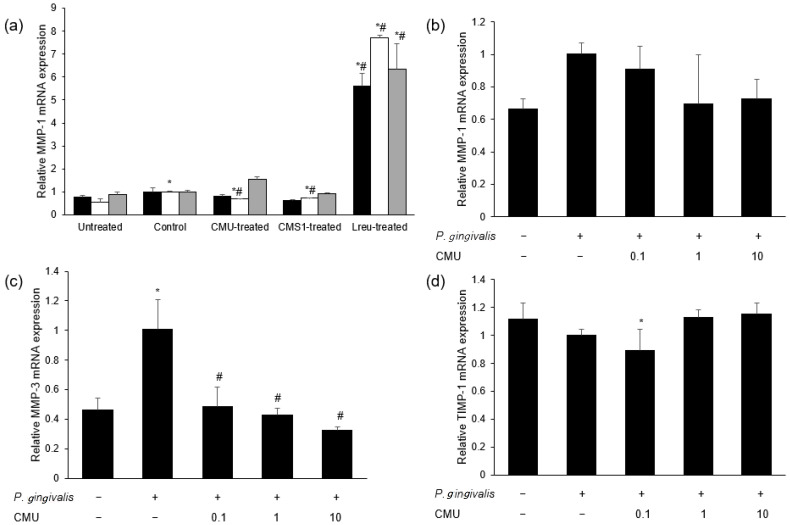
Effects of oral probiotics on the expression of MMPs (**a**–**c**) and TIMP-1 (**d**) induced by periodontopathogens. (**a**) Comparison of the effects of oral probiotics on the mRNA expression of MMP-1. Black bar, *P. gingivalis*; white bar, *F. nucleatum*; gray bar, *P. intermedia;* CMU, *W. cibaria* CMU; CMS1, *W. cibaria* CMS1; Lreu, *L. reuteri*. (**b**–**d**) Dose-dependent effects of *W. cibaria* CMU on the mRNA expression of MMP-1, MMP-3, and TIMP-1 induced by *P. gingivalis.* HGFs were pre-treated with *W. cibaria* CMU for 30 min at various doses (0.1, 1, and 10) and then incubated with *P. gingivalis* for 4 h. Data are presented as the mean ± standard deviation. * *p* < 0.05, compared to the untreated group. ^#^ *p* < 0.05, compared to the control group.

**Figure 7:**
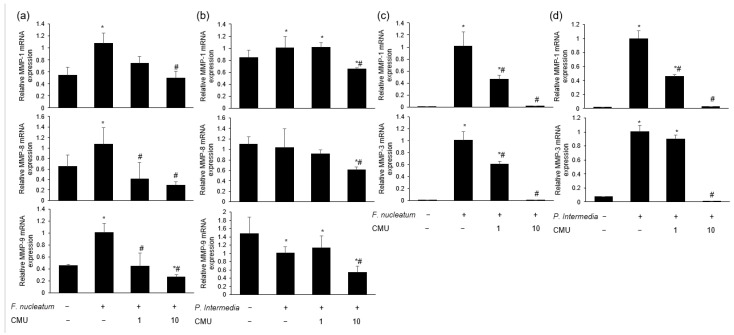
Dose-dependent effects of *W. cibaria* CMU on the mRNA expression of MMPs induced by *F. nucleatum* (**a**,**c**) and *P. intermedia* (**b**,**d**). HGFs were pre-treated with *W. cibaria* CMU for 30 min at doses of 1- and 10-times that of *F. nucleatum* or *P. intermedia* and then incubated with each periodontopathogen for 4 h (**a**,**b**) or 24 h (**c**,**d**). CMU, *W. cibaria* CMU. Data are presented as the mean ± standard deviation. * *p* < 0.05, compared to the untreated group. ^#^ *p* < 0.05, compared to the control group.

**Figure 8:**
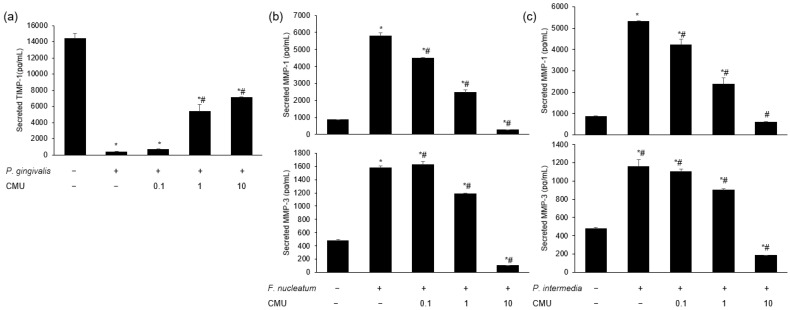
Dose-dependent effects of *W. cibaria* CMU on the protein expression of TIMP-1, MMP-1, and MMP-3 induced by *P. gingivalis* (**a**), *F. nucleatum* (**b**), and *P. intermedia* (**c**). HGFs were pre-treated with *W. cibaria* CMU for 30 min at various doses (0.1, 1, and 10) and then incubated with *P. gingivalis*, *F. nucleatum*, or *P. intermedia* (MOI = 100) for 24 h. CMU, *W. cibaria* CMU. Data are presented as the mean ± standard deviation. * *p* < 0.05, compared to the untreated group. ^#^ *p* < 0.05, compared to the control group.

*L. reuteri* stimulation increased the mRNA expression of MMP-1 compared to the treatment with periodontal bacteria alone (*p* < 0.05) (Figure 6a). We measured the mRNA expression of MMPs in HGFs pre-treated with various concentrations of *W. cibaria* and stimulated by each periodontopathogen. *W. cibaria* CMU altered the mRNA expression of MMP-1, MMP-3, and TIMP-1 in a dose-dependent manner compared to *P. gingivalis* treatment alone (Figure 6b–d). *W. cibaria* CMU also suppressed the mRNA expression of MMP-1, MMP-8, and MMP-9 in a dose-dependent manner compared to *F. nucleatum* or *P. intermedia* alone (*p* < 0.05) (Figure 7a,b). After stimulation with *F. nucleatum* or *P. intermedia* for 24 h, *W. cibaria* CMU suppressed the mRNA expression of MMP-1 and MMP-3 in a dose-dependent manner (*p* < 0.05) (Figure 7c,d). Pre-treatment with *W. cibaria* CMU increased TIMP-1 protein levels in a dose-dependent manner in HGFs infected with *P. gingivalis* (Figure 8a). *W. cibaria* CMU reduced the protein levels of MMP-1 and MMP-3 induced by *F. nucleatum* or *P. intermedia* in a dose-dependent manner. After stimulation with *F. nucleatum* and *P. intermedia* for 24 h, high concentrations of *W. cibaria* CMU reduced MMP-1 and MMP-3 protein levels by 95.4% and 98.2% (Figure 8b) as well as 88.9% and 96.5%, respectively (*p* < 0.05) (Figure 8c).

#### 3.3.5. Immunomodulatory Mechanisms of *W. cibaria*

**Figure 9:**
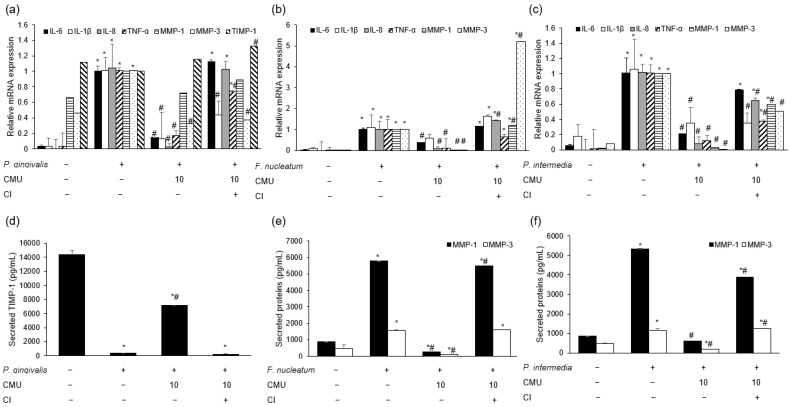
Effects of blocking direct contact between *W. cibaria* CMU and HGFs cells on the gene (**a**–**c**) and protein expression (**d**–**f**) of inflammatory mediators induced by *P. gingivalis* (**a**,**d**), *F. nucleatum* (**b**,**e**), and *P. intermedia* (**c**,**f**). *W. cibaria* CMU was pre-treated for 30 min at 10-fold the dose of *P. gingivalis*, *F. nucleatum*, or *P. intermedia* and then incubated with each periodontopathogen (MOI = 100) for 4 h (**a**–**c**) or 24 h (**d**–**f**). CMU, *W. cibaria* CMU; CI, cell culture inserts in the Transwell system. Data are presented as the mean ± standard deviation. * *p* < 0.05, compared to the untreated group. ^#^ *p* < 0.05, compared to the control group.

We investigated the effect of direct contact between *W. cibaria* CMU and HGFs on the regulation of periodontopathogen-induced inflammation by separating *W. cibaria* CMU from HGFs and periodontal bacteria using cell culture inserts in a Transwell system. After separating *W. cibaria* CMU, no inhibition of mRNA expression (Figure 9a–c) or protein secretion (Figure 9d–f) was observed for most of the inflammatory mediators.

## 4. Discussion

Probiotics are beneficial bacteria that provide various health benefits to the host [39]. Traditionally, probiotics have been used to improve gut health, especially for the treatment or prevention of gastrointestinal infections and diseases. The mechanisms of the action of probiotics include competitive adhesion inhibition, coaggregation, growth inhibition, bacteriocin production, and immune regulation [19]. Recent studies have proposed that probiotics can improve the regulation of body fat [48], vaginal health [49], and oral health [22]. In particular, many studies have reported effective strategies for the prevention and treatment of oral diseases using probiotics, including tooth decay and periodontal disease [22,30,32,34].

For decades, *S. mutans*, which ferments sugar, has been considered a major cause of dental caries [5] and has been considered to play an integral role in the etiology and pathogenesis of dental caries. However, recent DNA- and RNA-based studies have reported that *S. mutans* constitutes only a tiny fraction of the highly diverse bacterial community in carious lesions [50]. Therefore, in consideration of the polymicrobial nature of dental caries, a paradigm shift is needed for the pathogenesis of dental caries. Probiotics have been suggested to prevent dental caries by inhibiting *S. mutans* activity [23,28]. However, the supporting evidence for this is weak and it has been reported that probiotic bacterial strains may themselves be cariogenic. For example, *L. rhamnosus* GG, a well-known probiotic bacterium, was found to contribute to rather than inhibit the development of caries in experiments using dental tissue [51].

The current study is the first to confirm the inhibitory effect of oral probiotics against biofilm formation by *S. mutans* on artificial surfaces. We first compared the inhibitory effects of strains isolated from commercially available oral probiotic products against the growth of *S. mutans*. Using orthodontic wire as a surface for bacterial growth, we found that *L. reuteri, W. cibaria* CMU, and CMS1 had the most potent inhibitory effect, whereas *L. paracasei* and *L. gasseri* had no effect. Using artificial teeth as a surface for bacterial growth, we found that except for *W. cibaria* CMU and CMS1, *L. reuteri*, *L. paracasei*, *L. gasseri*, and *S. salivarius* caused a high degree of tooth discoloration similar to that in cultures with *S. mutans* alone, indicating no inhibitory effect. Several studies contradict our findings. Many studies have reported that *L. reuteri* inhibits biofilm formation by *S. mutans*, but most considered the effect of bacterial CFSs [23,24,32]. In our study, live *L. reuteri* was shown to inhibit *S. mutans*-induced biofilm formation on an orthodontic wire, but not on artificial teeth. Several strains of *L. paracasei* have also been reported to inhibit *S. mutans* biofilm formation [52,53]. Mann et al. [54] reported that *L. gasseri* inhibited the formation of biofilm by *S. mutans* on an orthodontic wire. Moreover, it has been reported that *S. salivarius* can respond to plaque formation and salivary acidity by producing dextranase and urease after colonizing the oral mucosa of humans [28].

Our study showed that *W. cibaria* CMU and CMS1 decreased the number of *S. mutans* cells in a dose-dependent manner. When cultured with *S. mutans* at the same dose, both *W. cibaria* CMU and CMS1 reduced the number of *S. mutans* by 4.1 log CFU/mL; even at a dose 100-times lower than that of *S. mutans*, *W. cibaria* CMU and CMS1 reduced the number of *S. mutans* by 3.4 and 3.5 log CFU/mL, respectively (Table 1). Consistent with our initial results but contrary to expectations, *L. reuteri*, *L. paracasei*, *L. gasseri*, and *S. salivarius* strains did not show inhibitory activity against *S. mutans* biofilm and by extension the development of caries.

Several factors are involved in periodontitis. In particular, the highly complex periodontal microbiome is known to play an important role not only in the initiation but also in the progression and establishment of periodontal disease [6,7,8,9,10,11]. Periodontitis results from dysbiosis of the periodontal microbiome, leading to changes in host–microbe crosstalk and initiation of the inflammatory response. *P. gingivalis* is a key pathogen in the development of periodontitis, along with several major complexes, including *F. nucleatum* and *P. intermedia* [6,7,8].

The pathogenic potential of plaque-causing bacteria has been demonstrated in their ability to produce many toxic substances, such as endotoxins, cell wall mucopeptides, fatty and organic acids, hydrogen sulfide, ammonia, indoles, amines, and leukotoxins [8]. Upon stimulation by these pathogens, pro-inflammatory cytokines are released from host cells to recruit immune cells. However, excessive inflammation results in tissue damage, bone resorption, and ultimately, tooth loss. Thus, the modulation of inflammation is a promising strategy for inhibiting disease progression. HGFs are the most abundant cells in gingival connective tissue and are common in periodontal tissues [55]. HGFs stimulated with pathogens have been reported to upregulate the gene expression of the pro-inflammatory cytokines IL-6, IL-1β, IL-8, and TNF-α, which facilitates the inflammatory cascade in periodontitis [10,11,55].

To confirm the preventive effect of oral probiotics on the impact of periodontal pathogens, we conducted line tests using *L. reuteri*, *W. cibaria* CMU, and CMS1 against periodontopathogens. After pre-treatment of HGFs with these oral probiotics, we found that the expression of IL-6, IL-1β, IL-8, and TNF-α genes induced by the periodontopathogens *P. gingivalis*, *F. nucleatum*, and *P. intermedia* were reduced by the *W. cibaria* strains, whereas *L. reuteri* upregulated their gene expression (Figure 4). We confirmed that *W. cibaria* CMU had a dose-dependent effect on the mRNA expression of pro-inflammatory cytokines, compared to the effect of *P. gingivalis* alone, especially at higher doses. In response to *F. nucleatum* and *P. intermedia* stimulation, *W. cibaria* CMU reduced the gene expression of most pro-inflammatory cytokines in a dose-dependent manner (except for that of IL-1β), especially at higher doses.

Periodontal lesions are characterized by excessive destruction of gingival connective tissue due to collagen degradation. MMPs secreted by HGFs are involved in the degradation of the extracellular matrix and bone collagen matrix [9,12,13]. Although various MMPs, including MMP-1, -3, -8, and -9, are involved in periodontal tissue remodeling [13], MMP-1 and MMP-3 play a particularly important role because collagen types I and III are predominant in periodontal connective tissues [14]. MMP-3 is expressed in a variety of cells other than gingival fibroblasts, including monocytes, endothelial cells, chondrocytes, and synovial cells, and is known to destroy connective tissue in chronic inflammatory diseases, such as periodontitis, rheumatoid arthritis, and osteoarthritis [13,14]. TIMPs regulate MMP activity, and among the four types of TIMPs, TIMP-1 exerts a strong inhibitory effect on fibroblast-derived MMPs [56].

In the present study, the expression of MMP-1 and -3 genes was increased by stimulation with *P. gingivalis*, *F. nucleatum*, and *P. intermedia*. *W. cibaria* CMU downregulated the expression of the genes MMP-1 and -3 in a dose-dependent manner, though the degree of downregulation differed slightly between periodontal pathogens. The production of TIMP-1 was decreased by stimulation with *P. gingivalis*, which was reversed by treatment with *W. cibaria* CMU in a dose-dependent manner. These results suggest that *W. cibaria* CMU regulates inflammation by upregulating TIMP-1 and downregulating MMP-3 gene expression induced by *P. gingivalis* in HGFs.

*L. reuteri* is known to inhibit the growth of various periodontal bacteria, including *P. gingivalis*, *F. nucleatum*, and *P. intermedia* through the production of reuterin, a bacteriocin [21]. Numerous investigations have also shown that several *L. reuteri* strains exert their immunomodulatory activity by reducing IL-6, IL-8, and TNF-α levels [22]. *L. reuteri* may alleviate the inflammatory response and reduce periodontal tissue destruction by regulating the imbalance between MMPs and TIMPs or reducing the production of pro-inflammatory cytokines such as TNF-α and IL-1β [25,26]. Along with *W. cibaria* CMU and CMS1, we showed that live *L. reuteri*, a commercial oral probiotic, showed antibacterial activity against *P. gingivalis*. However, this strain increased the gene expression of *P. gingivalis*-induced pro-inflammatory cytokines (IL-6, IL-1β, and IL-8) and MMP-1 in gingival cells, as well as in the gene expression of most *F. nucleatum*- and *P. intermedia*-induced pro-inflammatory cytokines and MMP-1. Therefore, *L. reuteri* did not have any inhibitory effects on the formation of caries and perio pathogens, indicating that the action of these probiotics is strain-specific.

Oral probiotics are known to function by colonizing the oral cavity and improving the microbiotic balance of the oral environment [38]. Therefore, commercialized products are mainly manufactured in a tablet or lozenge form that is optimized to exert its effects in the oral cavity over an extended period of time. To develop effective probiotics against periodontitis, it is also important to determine whether their anti-inflammatory effects are exerted through direct contact with gingival cells. Previous studies have shown that *W. cibaria* CMU requires direct contact with oral epithelial cells to inhibit *F. nucleatum*-induced IL-6 and IL-8 production [31]. In the present study, when using cell culture inserts, *W. cibaria* CMU was unable to inhibit the gene expression of pro-inflammatory cytokines and MMPs induced by periodontal pathogens when it was not in direct contact with HGFs. In addition, *W. cibaria* CMU failed to upregulate TIMP-1 production induced by *P. gingivalis* and did not inhibit MMP-1 and MMP-3 production induced by *F. nucleatum* and *P. intermedia*. Through this experiment, we confirmed that oral probiotics required direct contact with HGFs to exert their anti-inflammatory function, thereby regulating local inflammation by acting directly on gingival cells.

Further studies are needed for *W. cibaria* CMU to modulate the expression of protein mediators of inflammatory responses and signaling pathways. Moreover, the use of biomimetic hydroxyapatite and oral probiotics to reduce the incidence of DMFT (Decayed Missing Filled Teeth) and periodontal risk, as well as the use of ozone and photodynamic therapy to reduce the bacterial load, is yet to be explored [57].

## 5. Conclusions

This study is the first to confirm that the oral probiotic *W. cibaria* CMU inhibits biofilm formation by *S. mutans* by using an artificial tooth model. In addition, this probiotic strain was shown to act directly on gingival tissue cells to inhibit the gene expression of pro-inflammatory cytokines and MMPs induced by periodontal bacteria. These results underscore the potential use of the oral probiotic *W. cibaria* CMU in the proactive action against the incidence of oral diseases such as dental caries and periodontitis.

## Figures and Tables

**Table 1:** Effects of oral probiotics on the growth of *S. mutans* under tube wire test conditions.

Group	*S. mutans* (CFU/mL)	Reduction (Log CFU/mL)	% Growth Inhibition
*S. mutans* alone	3.52 × 10^9^ ± 7.66 × 10^8 d^	-	-
*S. mutans* + *L. reuteri* (1:1)	2.27 × 10^9^ ± 5.77 × 10^7 b^	0.2	35.61
*S. mutans* + *L. paracasei* (1:1)	3.07 × 10^9^ ± 1.53 × 10^8 c^	0.1	12.88
*S. mutans* + *L. gasseri* (1:1)	1.67 × 10^8^ ± 5.86 × 10^7 a^	1.3	95.27
*S. mutans* + *S. salivarius* (1:1)	1.20 × 10^8^ ± 3.61 × 10^7 a^	1.5	96.59
*S. mutans* + *W. cibaria* CMU (1:0.01)	1.46 × 10^6^ ± 1.22 × 10^6 a^	3.4	99.96
*S. mutans* + *W. cibaria* CMU (1:0.1)	4.37 × 10^5^ ± 1.17 × 10^5 a^	3.9	99.99
*S. mutans* + *W. cibaria* CMU (1:1)	2.92 × 10^5^ ± 1.72 × 10^5 a^	4.1	99.99
*S. mutans* + *W. cibaria* CMS1 (1:0.01)	1.1.0 × 10^6^ ± 9.94 × 10^5 a^	3.5	99.97
*S. mutans* + *W. cibaria* CMS1 (1:0.1)	7.30 × 10^5^ ± 5.42 × 10^5 a^	3.7	99.98
*S. mutans* + *W. cibaria* CMS1 (1:1)	2.92 × 10^5^ ± 1.64 × 10^5 a^	4.1	99.99

Data are presented as the mean ± standard deviation. Different letters indicate significant differences at *p* < 0.05.

**Table 2:** Cell viability of HGFs treated with oral probiotics.

Group	Cell Viability (% of Control)	
	Only DMEM	DMEM + 2% FBS
Untreated control	100.0 ± 4.4	100.0 ± 4.1
*L. reuteri* (MOI = 0.1)	115.2 ± 12.7	84.0 ± 23.9
*L. reuteri* (MOI = 1)	122.5 ± 11.5	89.0 ± 25.6
*L. reuteri* (MOI = 10)	121.6 ± 5.0	86.5 ± 6.0
*L. reuteri* (MOI = 100)	127.6 ± 8.1	92.0 ± 17.4
*W. cibaria* CMU (MOI = 0.1)	108.4 ± 11.8	100.5 ± 12.2
*W. cibaria* CMU (MOI = 1)	119.5 ± 9.7	103.1 ± 17.4
*W. cibaria* CMU (MOI = 10)	118.6 ± 14.4	92.7 ± 11.5
*W. cibaria* CMU (MOI = 100)	166.8 ± 12.2	112.8 ± 10.9
*W. cibaria* CMS1 (MOI = 0.1)	111.4 ± 2.7	87.7 ± 4.8
*W. cibaria* CMS1 (MOI = 1)	127.0 ± 4.2	85.4 ± 2.4
*W. cibaria* CMS1 (MOI = 10)	128.0 ± 3.6	87.3 ± 2.8
*W. cibaria* CMS1 (MOI = 100)	153.4 ± 6.3	100.8 ± 3.1

Data are presented as the mean ± standard deviation. MOI, multiplicity of infection.

## Data Availability

The data presented in this study are available upon request from the corresponding author.

## Supplementary Materials

The following supporting information can be downloaded at: https://www.mdpi.com/article/10.3390/microorganisms11040962/s1, Table S1: DPPH radical scavenging activities (%) of oral probiotics.

## References

[1] Kassebaum N.J., Bernabé E., Dahiya M., Bhandari B., Murray C.J., Marcenes W. (2015). Global burden of untreated caries: A systematic review and metaregression. J. Dent. Res..

[2] Kassebaum N.J., Bernabé E., Dahiya M., Bhandari B., Murray C.J., Marcenes W. (2014). Global burden of severe periodontitis in 1990–2010: A systematic review and meta-regression. J. Dent. Res..

[3] Wolf T.G., Cagetti M.G., Fisher J.M., Seeberger G.K., Campus G. (2021). Non-communicable diseases and oral health: An overview. Front. Oral Health.

[4] Butera A., Maiorani C., Morandini A., Simonini M., Morittu S., Trombini J., Scribante A. (2022). Evaluation of children caries risk factors: A narrative review of nutritional aspects, oral hygiene habits, and bacterial alterations. Children.

[5] Lemos J.A., Palmer S.R., Zeng L., Wen Z.T., Kajfasz J.K., Freires I.A., Abranches J., Brady L.J. (2019). The Biology of *Streptococcus mutans*. Microbiol. Spectr..

[6] Kinane D.F. (2001). Causation and pathogenesis of periodontal disease. Periodontology 2000.

[7] Riep B., Edesi-Neuss L., Claessen F., Skarabis H., Ehmke B., Flemmig T.F., Bernimoulin J.P., Göbel U.B., Moter A. (2009). Are putative periodontal pathogens reliable diagnostic markers?. J. Clin. Microbiol..

[8] Socransky S.S., Haffajee A.D. (1994). Evidence of bacterial etiology: A historical perspective. Periodontology 2000.

[9] Bodet C., Chandad F., Grenier D. (2006). Inflammatory responses of a macrophage/epithelial cell co-culture model to mono and mixed infections with *Porphyromonas gingivalis*, *Treponema denticola*, and *Tannerella forsythia*. Microbes Infect..

[10] Hoare A., Soto C., Rojas-Celis V., Bravo D. (2019). Chronic inflammation as a link between periodontitis and carcinogenesis. Mediat. Inflamm..

[11] Leite F.R.M., Nascimento G.G., Møller H.J., Belibasakis G.N., Bostanci N., Smith P.C., López R. (2022). Cytokine profiles and the dynamic of gingivitis development in humans. J. Clin. Periodontol..

[12] Gürkan A., Emingil G., Saygan B.H., Atilla G., Cinarcik S., Köse T., Berdeli A. (2007). Matrix metalloproteinase-2, -9, and -12 gene polymorphisms in generalized aggressive periodontitis. J. Periodontol..

[13] Loo W.T., Wang M., Jin L.J., Cheung M.N., Li G.R. (2011). Association of matrix metalloproteinase (MMP-1, MMP-3 and MMP-9) and cyclooxygenase-2 gene polymorphisms and their proteins with chronic periodontitis. Arch. Oral. Biol..

[14] Domeij H., Yucel-Lindberg T., Modéer T. (2002). Signal pathways involved in the production of MMP-1 and MMP-3 in human gingival fibroblasts. Eur. J. Oral Sci..

[15] Bollen C.M., Quirynen M. (1996). Microbiological response to mechanical treatment in combination with adjunctive therapy. A review of the literature. J. Periodontol..

[16] Page R.C. (2004). The microbiological case for adjunctive therapy for periodontitis. J. Int. Acad. Periodontol..

[17] Sanders W.E. (1988). Efficacy, safety, and potential economic benefits of oral ciprofloxacin in the treatment of infections. Rev. Infect. Dis..

[18] Heelan J.S., Hasenbein M.E., McAdam A.J. (2004). Resistance of group B *streptococcus* to selected antibiotics, including erythromycin and clindamycin. J. Clin. Microbiol..

[19] Aliabadi T., Saberi E.A., Tabatabaei A.M., Tahmasebi E. (2022). Antibiotic use in endodontic treatment during pregnancy: A narrative review. Eur. J. Transl. Myol..

[20] Devine D.A., Marsh P.D., Meade J. (2015). Modulation of host responses by oral commensal bacteria. J. Oral. Microbiol..

[21] Jansen P.M., Abdelbary M.M.H., Conrads G. (2021). A concerted probiotic activity to inhibit periodontitis-associated bacteria. PLoS ONE.

[22] Zhang Y., Ding Y., Guo Q. (2022). Probiotic species in the management of periodontal diseases: An overview. Front. Cell Infect. Microbiol..

[23] Söderling E.M., Marttinen A.M., Haukioja A.L. (2011). Probiotic lactobacilli interfere with *Streptococcus mutans* biofilm formation in vitro. Curr. Microbiol..

[24] Kang M.S., Oh J.S., Lee H.C., Lim H.S., Lee S.W., Yang K.H., Choi N.K., Kim S.M. (2011). Inhibitory effect of *Lactobacillus reuteri* on periodontopathic and cariogenic bacteria. J. Microbiol..

[25] Szkaradkiewicz A.K., Stopa J., Karpiński T.M. (2014). Effect of oral administration involving a probiotic strain of *Lactobacillus reuteri* on pro-inflammatory cytokine response in patients with chronic periodontitis. Arch. Immunol. Ther. Exp..

[26] İnce G., Gürsoy H., İpçi Ş.D., Cakar G., Emekli-Alturfan E., Yılmaz S. (2015). Clinical and biochemical evaluation of lozenges containing *Lactobacillus reuteri* as an adjunct to non-surgical periodontal therapy in chronic periodontitis. J. Periodontol..

[27] Cosseau C., Devine D.A., Dullaghan E., Gardy J.L., Chikatamarla A., Gellatly S., Yu L.L., Pistolic J., Falsafi R., Tagg J. (2008). The commensal *Streptococcus salivarius* K12 downregulates the innate immune responses of human epithelial cells and promotes host-microbe homeostasis. Infect. Immun..

[28] Heng N.C., Haji-Ishak N.S., Kalyan A., Wong A.Y., Lovric M., Bridson J.M., Artamonova J., Stanton J.A., Wescombe P.A., Burton J.P. (2011). Genome sequence of the bacteriocin-producing oral probiotic *Streptococcus salivarius* strain M18. J. Bacteriol..

[29] MacDonald K.W., Chanyi R.M., Macklaim J.M., Cadieux P.A., Reid G., Burton J.P. (2021). *Streptococcus salivarius* inhibits immune activation by periodontal disease pathogens. BMC Oral Health.

[30] Kang M.S., Chung J., Kim S.M., Yang K.H., Oh J.S. (2006). Effect of *Weissella cibaria* isolates on the formation of *Streptococcus mutans* biofilm. Caries Res..

[31] Kang M.S., Lim H.S., Kim S.M., Lee H., Oh J.S. (2011). Effect of *Weissella cibaria* on *Fusobacterium nucleatum*-induced interleukin-6 and interleukin-8 production in KB cells. J. Bacteriol. Virol..

[32] Jang H.J., Kang M.S., Yi S.H., Hong J.Y., Hong S.P. (2016). Comparative study on the characteristics of *Weissella cibaria* CMU and probiotic strains for oral care. Molecules.

[33] Kang M.S., Yeu J.E., Oh J.S., Shin B.A., Kim J.H. (2017). Complete genome sequences of *Weissella cibaria* strains CMU, CMS1, CMS2, and CMS3 isolated from infant saliva in South Korea. Genome Announc..

[34] Do K.H., Park H.E., Kang M.S., Kim J.T., Yeu J.E., Lee W.K. (2019). Effects of *Weissella cibaria* CMU on halitosis and calculus, plaque, and gingivitis indices in beagles. J. Vet. Dent..

[35] Kang M.S., Lee D.S., Lee S.A., Kim M.S., Nam S.H. (2020). Effects of probiotic bacterium *Weissella cibaria* CMU on periodontal health and microbiota: A randomised, double-blind, placebo-controlled trial. BMC Oral Health.

[36] Kang M.S., Lee D.S., Kim M., Lee S.A., Nam S.H. (2021). A randomized, double-blind, placebo-controlled trial to assess the acidogenic potential of dental biofilms through a tablet containing *Weissella cibaria* CMU. Int. J. Environ. Res. Public Health.

[37] Kim M.J., You Y.O., Kim H.J. (2022). Heat-inactivated *Weissella cibaria* CMU downregulates the mRNA expression of proinflammatory cytokines and matrix metalloproteinases in *Porphyromonas gingivalis* lipopolysaccharide-stimulated human gingival fibroblasts. Korean J. Oral Anatomy.

[38] Han H.S., Yum H., Cho Y.D., Kim S. (2023). Improvement of halitosis by probiotic bacterium *Weissella cibaria* CMU: A randomized controlled trial. Front Microbiol..

[39] Reid G. (2005). Food and Agricultural Organization of the United Nation and the WHO. The importance of guidelines in the development and application of probiotics. Curr. Pharm. Des..

[40] Salminen S., Collado M.C., Endo A., Hill C., Lebeer S., Quigley E.M.M., Sanders M.E., Shamir R., Swann J.R., Szajewska H. (2021). The International Scientific Association of Probiotics and Prebiotics (ISAPP) consensus statement on the definition and scope of postbiotics. Nat. Rev. Gastroenterol. Hepatol..

[41] Butera A., Gallo S., Pascadopoli M., Taccardi D., Scribante A. (2022). Home oral care of periodontal patients using antimicrobial gel with postbiotics, lactoferrin, and aloe barbadensis leaf juice powder vs. conventional chlorhexidine gel: A split-mouth randomized clinical trial. Antibiotics.

[42] Butera A., Gallo S., Pascadopoli M., Maiorani C., Milone A., Alovisi M., Scribante A. (2022). Paraprobiotics in non-surgical periodontal therapy: Clinical and microbiological aspects in a 6-month follow-up domiciliary protocol for oral hygiene. Microorganisms.

[43] Butera A., Gallo S., Maiorani C., Molino D., Chiesa A., Preda C., Esposito F., Scribante A. (2020). Probiotic Alternative to Chlorhexidine in Periodontal Therapy: Evaluation of Clinical and Microbiological Parameters. Microorganisms.

[44] Babaahmady K.G., Challacombe S.J., Marsh P.D., Newman H.N. (1998). Ecological study of *Streptococcus mutans, Streptococcus sobrinus* and *Lactobacillus* spp. at sub-sites from approximal dental plaque from children. Caries Res..

[45] Yang Y., Park B.I., Hwang E.H., You Y.O. (2016). Composition analysis and inhibitory effect of *Sterculia lychnophora* against biofilm formation by *Streptococcus mutans*. Evid. Based Complement. Alternat. Med..

[46] Khalfallah G., Gartzen R., Möller M., Heine E., Lütticken R. (2021). A new approach to harness probiotics against common bacterial skin pathogens: Towards living antimicrobials. Probiotics Antimicrob. Proteins.

[47] De Marco S., Sichetti M., Muradyan D., Piccioni M., Traina G., Pagiotti R., Pietrella D. (2018). Probiotic cell-free supernatants exhibited anti-inflammatory and antioxidant activity on human gut epithelial cells and macrophages stimulated with LPS. Evid. Based Complement. Alternat. Med..

[48] Koutnikova H., Genser B., Monteiro-Sepulveda M., Faurie J.M., Rizkalla S., Schrezenmeir J., Clément K. (2019). Impact of bacterial probiotics on obesity, diabetes and non-alcoholic fatty liver disease related variables: A systematic review and meta-analysis of randomised controlled trials. BMJ Open.

[49] Mei Z., Li D. (2022). The role of probiotics in vaginal health. Front. Cell Infect. Microbiol..

[50] Simón-Soro A., Mira A. (2015). Solving the etiology of dental caries. Trends Microbiol..

[51] Schwendicke F., Dörfer C., Kneist S., Meyer-Lueckel H., Paris S. (2014). Cariogenic effects of probiotic *Lactobacillus rhamnosus* GG in a dental biofilm model. Caries Res..

[52] Tanzer J.M., Thompson A., Lang C., Cooper B., Hareng L., Gamer A., Reindl A., Pompejus M. (2010). Caries inhibition by and safety of *Lactobacillus paracasei* DSMZ16671. J. Dent. Res..

[53] de Alvarenga J.A., de Barros P.P., de Camargo Ribeiro F., Rossoni R.D., Garcia M.T., Dos Santos Velloso M., Shukla S., Fuchs B.B., Shukla A., Mylonakis E. (2021). Probiotic Effects of *Lactobacillus paracasei* 28.4 to inhibit *Streptococcus mutans* in a gellan-based formulation. Probiotics Antimicrob. Proteins.

[54] Mann S., Park M.S., Johnston T.V., Ji G.E., Hwang K.T., Ku S. (2021). Oral probiotic activities and biosafety of *Lactobacillus gasseri* HHuMIN D. Microb. Cell Fact..

[55] Naruishi K. (2022). Biological roles of fibroblasts in periodontal diseases. Cells.

[56] Gomez D.E., Alonso D.F., Yoshiji H., Thorgeirsson U.P. (1997). Tissue inhibitors of metalloproteinases: Structure, regulation and biological functions. Eur. J. Cell Biol..

[57] Butera A., Maiorani C., Gallo S., Pascadopoli M., Venugopal A., Marya A., Scribante A. (2022). Evaluation of adjuvant systems in non-surgical peri-implant treatment: A literature review. Healthcare.

