# Supplementary material for: In Vitro Preventive Effect and Mechanism of Action of Weissella cibaria CMU against Streptococcus mutans Biofilm Formation and Periodontal Pathogens
Source: Microorganisms. 2023 Apr 7;11(4):962. doi: 10.3390/microorganisms11040962 (PMC10146839; doi:10.3390/microorganisms11040962)
Supplement: Supplementary file 1 [file microorganisms-11-00962-s001.zip › microorganisms-2323132-supplementary.pdf]

Table S1. 2,2-diphenyl-1-picrylhydrazyl (DPPH) radical scavenging activities (%) of oral probiotics (Raw data)

OD_{517}				DPPH radical scavenging activities (%)		
MRS blank	*W. cibaria* CMU	*W. cibaria* CMS1	*L. reuteri*	*W. cibaria* CMU	*W. cibaria* CMS1	*L. reuteri*
0.181	0.136	0.133	0.135	24.7	26.5	25.5
0.163	0.136	0.137	0.135	16.4	15.9	16.8
0.158	0.137	0.144	0.162	22.9	12.9	5.6
0.157	0.132	0.139	0.142	15.9	11.7	13.0
